# Supplementary material for: Automatic vectorization of historical maps: A benchmark
Source: PLoS One. 2024 Feb 15;19(2):e0298217. doi: 10.1371/journal.pone.0298217 (PMC10868791; doi:10.1371/journal.pone.0298217)
Supplement: S1 Appendix — Full list of experimental results. (PDF) [file pone.0298217.s001.pdf]

Table 11. Training parameters for each model. Additionally, for each model we use a plateau scheduler, and a weigh decay of $2 \cdot 10^{-4}$.
For architectures: ^{1}: trained from scratch, ^{2}: trained using pre-trained weights, $^{1+2}$: both trainings.

Architecture	Learning rate	Optimizer	#Param.
(mini-)U-Net^1	$1 \cdot 10^{-4}$	ADAM	4M, 17M
HED^{1+2}	$1 \cdot 10^{-4}$	ADAM	138M
BDCN^{1+2}	$1 \cdot 10^{-4}$	ADAM	139M
U-Net+BALoss^2	$1 \cdot 10^{-4}$	ADAM	17M
U-Net+Topoloss^2	$1 \cdot 10^{-5}$	SGD	17M
U-Net+Mosin^2	$1 \cdot 10^{-4}$	SGD	17M
ViT^2	$1 \cdot 10^{-5}$	ADAMW	88M
PVT^2	$1 \cdot 10^{-4}$	ADAM	70M
Deep Watershed^1	$1 \cdot 10^{-5}$	ADAM	35M

A Appendix

We provide here extra details about the networks used, as well as the full list of experimental results.

A.1 Deep edge filters and training parameters

The details about the architectures used for deep edge filtering are provided in table 11. It is worth noting that the U-Net architecture not only achieves a great accuracy, but also is very efficient.

A.2 Full list of experimental results

The complete list of results presented along this paper, as well as some extra results, are presented in the following tables.

- Table 12 details the results for all the variants which use a **naive connected component labelling** for the closed shape extraction stage.
- Table 13 details the results for all the variants which use **some watershed variant** for the closed shape extraction stage, with the **deep edge filter parameter selection method of [5]** (retain the deep edge filter with the best performance on the validation set after a simple binarization of the edge probability map ($\theta = 0.5$) followed by connected component labelling).
- Table 14 details the results for all the variants which use **some watershed variant** for the closed shape extraction stage, while **selecting the deep edge filter parameters using a joint optimization**, as proposed in this paper.

Table 12. COCO Panoptic scores on validation and test set for all variants under test, using naive connected component labelling as CSE. For the architectures, * indicate pre-trained variants.

DEF					DEF selection	CSE		Evaluation					
Archi.	Training config.	Loss function	Augmentation			Method	Param.	Val. set			Test set		
			Contrast streching	Geometric transform			θ	PQ	SQ	RQ	PQ	SQ	RQ
U-Net	Original	binxent	no	none	CC@0.5	CC@best	0.9	34.8	80.5	43.3	8.1	78.2	10.4
HED	Original	binxent	no	none	CC@0.5	CC@best	0.3	23.2	76.5	30.3	14.0	74.8	18.8
HED*	Original	binxent	no	none	CC@0.5	CC@best	0.7	27.6	77.6	35.6	16.2	76.1	21.3
BDCN	Original	binxent	no	none	CC@0.5	CC@best	0.9	27.7	80.6	34.4	10.2	80.4	12.7
BDCN*	Original	binxent	no	none	CC@0.5	CC@best	0.8	27.6	82.1	33.7	8.9	82.8	10.7
U-Net	Proposed	binxent	no	none	CC@0.5	CC@0.5	0.5	46.8	87.5	53.5	41.2	85.4	48.2
mini U-Net	Proposed	binxent	no	none	CC@0.5	CC@0.5	0.5	51.8	87.2	59.3	31.3	81.4	38.4
ViT*	Proposed	binxent	no	none	CC@0.5	CC@0.5	0.5	29.9	80.5	37.1	28.8	77.5	37.2
PVT*	Proposed	binxent	no	none	CC@0.5	CC@0.5	0.5	35.2	85.0	41.4	25.4	81.7	31.1
U-Net*	Proposed	bal	no	none	CC@0.5	CC@0.5	0.5	58.0	87.6	66.1	43.4	84.4	51.4
U-Net*	Proposed	topo	no	none	CC@0.5	CC@0.5	0.5	56.8	87.7	64.8	30.4	85.7	35.5
U-Net*	Proposed	mosin	no	none	CC@0.5	CC@0.5	0.5	52.5	88.7	59.2	18.9	86.9	21.7
HED	Proposed	binxent	no	none	CC@0.5	CC@0.5	0.5	52.2	86.8	60.2	42.7	85.2	50.1
HED*	Proposed	binxent	no	none	CC@0.5	CC@0.5	0.5	32.4	87.0	37.3	44.5	85.2	52.3
BDCN	Proposed	binxent	no	none	CC@0.5	CC@0.5	0.5	51.4	86.5	59.5	43.4	85.2	50.9
BDCN*	Proposed	binxent	no	none	CC@0.5	CC@0.5	0.5	55.7	87.0	64.0	41.4	86.1	48.1
U-Net	Proposed	binxent	yes	none	CC@0.5	CC@0.5	0.5	40.5	87.8	46.1	35.3	85.8	41.2
U-Net	Proposed	binxent	no	Aff.	CC@0.5	CC@0.5	0.5	56.8	87.1	65.2	42.6	85.6	49.7
U-Net	Proposed	binxent	yes	Aff.	CC@0.5	CC@0.5	0.5	59.2	87.6	67.7	46.7	86.0	54.3
U-Net	Proposed	binxent	no	Hom.	CC@0.5	CC@0.5	0.5	58.4	86.8	67.3	45.8	85.3	53.7
U-Net	Proposed	binxent	yes	Hom.	CC@0.5	CC@0.5	0.5	60.0	86.9	69.0	48.5	85.2	56.9
U-Net	Proposed	binxent	no	TPS	CC@0.5	CC@0.5	0.5	57.8	87.8	65.8	38.8	86.5	44.9
U-Net	Proposed	binxent	yes	TPS	CC@0.5	CC@0.5	0.5	56.9	87.8	64.8	44.2	86.5	51.1
DWS	Proposed	binxent	no	none	CC@0.5	CC@0.5	0.5	54.0	87.4	61.7	28.5	84.9	33.5

Table 13. COCO Panoptic scores on validation and test set for all variants under test, using any watershed variant as CSE and DEF selection from [5]. For the architectures, * indicate pre-trained variants.

DEF					DEF selection	CSE			Evaluation					
Archi.	Training config.	Loss function	Augmentation			Method	Param.		Val. set			Test set		
			Contrast streching	Geometric transform			σ	δ	PQ	SQ	RQ	PQ	SQ	RQ
U-Net	Proposed	binxent	no	none	CC@0.5	MWS	50.0	8.0	59.8	87.7	68.2	46.7	86.9	53.7
mini U-Net	Proposed	binxent	no	none	CC@0.5	MWS	100.0	10.0	55.9	87.2	64.1	46.3	86.7	53.4
ViT*	Proposed	binxent	no	none	CC@0.5	MWS	500.0	10.0	38.5	80.9	47.6	34.9	80.5	43.4
PVT*	Proposed	binxent	no	none	CC@0.5	MWS	400.0	10.0	45.4	85.1	53.4	36.9	83.5	44.2
U-Net*	Proposed	bal	no	none	CC@0.5	MWS	50.0	6.0	62.3	87.3	71.3	44.1	87.0	50.7
U-Net*	Proposed	topo	no	none	CC@0.5	MWS	50.0	8.0	59.0	88.1	67.0	43.1	86.4	49.9
U-Net*	Proposed	mosin	no	none	CC@0.5	MWS	50.0	1.0	54.1	88.2	61.3	36.0	87.4	41.2
HED	Proposed	binxent	no	none	CC@0.5	MWS	400.0	10.0	47.5	86.8	54.7	41.0	85.2	48.1
HED*	Proposed	binxent	no	none	CC@0.5	MWS	400.0	10.0	51.5	87.5	58.9	43.9	86.2	50.9
BDCN	Proposed	binxent	no	none	CC@0.5	MWS	400.0	10.0	48.9	86.9	56.3	41.3	85.8	48.1
BDCN*	Proposed	binxent	no	none	CC@0.5	MWS	400.0	10.0	54.7	88.6	61.7	46.4	87.1	53.3
U-Net	Proposed	binxent	yes	none	CC@0.5	MWS	100.0	6.0	57.1	87.9	65.0	46.6	87.0	53.5
U-Net	Proposed	binxent	no	Aff.	CC@0.5	MWS	100.0	10.0	60.8	87.7	69.3	48.0	86.4	55.5
U-Net	Proposed	binxent	yes	Aff.	CC@0.5	MWS	100.0	9.0	60.6	88.1	68.8	51.0	87.1	58.6
U-Net	Proposed	binxent	no	Hom.	CC@0.5	MWS	300.0	10.0	57.9	88.2	65.7	48.5	87.0	55.7
U-Net	Proposed	binxent	yes	Hom.	CC@0.5	MWS	200.0	10.0	59.5	88.1	67.5	50.4	86.4	58.4
U-Net	Proposed	binxent	no	TPS	CC@0.5	MWS	100.0	10.0	59.4	88.1	67.4	47.5	87.0	54.6
U-Net	Proposed	binxent	yes	TPS	CC@0.5	MWS	100.0	10.0	59.2	88.0	67.3	50.1	86.9	57.7
U-Net	Original	binxent	no	none	CC@0.5	MWS	50.0	0.0	56.6	87.7	64.5	18.3	85.2	21.4
HED	Original	binxent	no	none	CC@0.5	MWS	200.0	10.0	50.5	87.2	57.9	35.6	84.6	42.0
HED*	Original	binxent	no	none	CC@0.5	MWS	300.0	8.0	52.8	87.6	60.3	38.4	85.5	44.9
BDCN	Original	binxent	no	none	CC@0.5	MWS	300.0	8.0	52.5	87.8	59.8	34.9	85.8	40.6
BDCN*	Original	binxent	no	none	CC@0.5	MWS	300.0	7.0	53.0	88.1	60.1	37.8	86.4	43.8

Table 14. COCO Panoptic scores on validation and test set for all variants under test, using any watershed variant as CSE and Joint Optimization for DEF selection. For the architectures, * indicate pre-trained variants.

DEF					DEF selection	CSE			Evaluation					
Archi.	Training config.	Loss function	Augmentation			Method	Param.		Val. set			Test set		
			Contrast streching	Geometric transform			σ	δ	PQ	SQ	RQ	PQ	SQ	RQ
U-Net	Proposed	binxent	no	none	JO	MWS	50.0	10.0	60.4	88.2	68.5	47.1	86.8	54.3
mini U-Net	Proposed	binxent	no	none	JO	MWS	100.0	10.0	56.7	87.7	64.6	45.1	86.0	52.5
ViT*	Proposed	binxent	no	none	JO	MWS	500.0	10.0	38.6	80.9	47.8	34.7	80.4	43.1
PVT*	Proposed	binxent	no	none	JO	MWS	400.0	9.0	45.7	85.4	53.5	36.6	83.0	44.2
U-Net*	Proposed	bal	no	none	JO	MWS	50.0	1.0	63.1	87.6	72.0	45.6	86.3	52.9
U-Net*	Proposed	topo	no	none	JO	MWS	100.0	6.0	59.9	88.1	68.0	36.9	84.2	43.8
U-Net*	Proposed	mosin	no	none	JO	MWS	50.0	1.0	57.7	88.3	65.3	36.0	87.4	41.2
HED	Proposed	binxent	no	none	JO	MWS	400.0	10.0	47.6	86.8	54.9	40.8	85.0	47.9
HED*	Proposed	binxent	no	none	JO	MWS	400.0	10.0	51.8	87.5	59.2	43.7	86.2	50.7
BDCN	Proposed	binxent	no	none	JO	MWS	400.0	10.0	49.1	86.9	56.5	41.1	86.0	47.8
BDCN*	Proposed	binxent	no	none	JO	MWS	400.0	9.0	55.0	88.5	62.1	47.0	87.3	53.8
U-Net	Proposed	binxent	yes	none	JO	MWS	100.0	6.0	57.3	88.2	65.0	47.2	86.7	54.4
U-Net	Proposed	binxent	no	Aff.	JO	MWS	100.0	9.0	61.0	87.9	69.4	47.7	86.5	55.1
U-Net	Proposed	binxent	yes	Aff.	JO	MWS	100.0	10.0	61.1	88.1	69.4	50.7	86.8	58.5
U-Net	Proposed	binxent	no	Hom.	JO	MWS	200.0	10.0	58.4	87.9	66.5	49.6	86.9	57.1
U-Net	Proposed	binxent	yes	Hom.	JO	MWS	200.0	10.0	59.5	88.2	67.4	50.4	86.7	58.2
U-Net	Proposed	binxent	no	TPS	JO	MWS	100.0	10.0	59.8	88.3	67.8	47.9	86.9	55.1
U-Net	Proposed	binxent	yes	TPS	JO	MWS	100.0	7.0	59.6	88.2	67.5	51.1	86.8	58.8
DWS	Proposed	binxent	no	none	JO	MWS	0.0	0.0	54.0	87.4	61.7	28.5	84.9	33.5
